# Whole genome sequencing reveals candidate causal genetic variants for spastic syndrome in Holstein cattle

**DOI:** 10.1038/s41598-024-82446-z

**Published:** 2024-12-28

**Authors:** Joana G.P. Jacinto, Anna Letko, Irene M. Häfliger, Eylem Emek Akyürek, Roberta Sacchetto, Arcangelo Gentile, Cord Drögemüller

**Affiliations:** 1 Institute of Genetics, Vetsuisse Faculty, University of Bern, Bern, 3012 Switzerland; 2 Clinic for Ruminants, Vetsuisse Faculty, University of Bern, Bern, 3012 Switzerland; 3 Department of Comparative Biomedicine and Food Science, University of Padua, Legnaro (Padua), 35020 Italy; 4 Department of Veterinary Medical Sciences, University of Bologna, Ozzano dell’Emilia, Bologna, 40064 Italy

**Keywords:** Animal breeding, Clinical genetics, Genomics, Medical genetics, Sequencing

## Abstract

Bovine spastic syndrome (SS) is a progressive, adult-onset neuromuscular disorder (NMD). SS is inherited but the mode of inheritance is unclear. The aim of this study was to characterize the phenotype and to identify a possible genetic cause of SS by whole-genome sequencing (WGS) and focusing on protein-changing variants. Seven affected unrelated Holstein cattle of both sexes were referred for SS at a mean age of 5.3 years (S.D.±1.1) showing intermittent spasm of the skeletal muscles of the pelvic girdle. Assuming monogenic recessive inheritance, analysis of the WGS data did not reveal any private variants common to all cases. Searching for homozygous rare variants considering each case individually, allowed the identification of a rare recessive likely pathogenic missense variant in *TOR3A* for one case with an allele frequency of 1.69% in a global Holstein population. In the remaining six SS cases, we identified seven potentially dominant de novo mutations or inherited alleles as private heterozygous, mostly missense, variants of uncertain significance involving seven different NMD candidate genes: *MPEG1*, *LHX8*, *WHAMM*, *NGRN*, *TTN*, *ATP1A1*, *PCDH1*. All eight candidate causal variants identified were predicted to be deleterious. This study describes for the first time WGS findings in confirmed cases of bovine SS and provides evidence for a heterogeneous genetic cause of SS in cattle.

**Supplementary Information:**

The online version contains supplementary material available at 10.1038/s41598-024-82446-z.

## Introduction

Genetic neuromuscular disorders (NMDs) are a heterogeneous group of conditions that can be congenital or occur later in life^1^. They affect muscles, motor neurons, peripheral nerves, or the neuromuscular junction^2^. NMDs are characterized by progressive muscle degeneration and weakness that primarily or secondarily affect skeletal muscle function^3^. In humans, NMDs are classified into 16 groups of disorders, including, among others, muscular dystrophies, myotonic syndromes, myopathies and, hereditary paraplegias (HPs)^4^. In addition, Mendelian forms of NMDs can follow a recessive, dominant and X-linked mode of inheritance (MOI) and have been associated with variants in more than 680 genes^1^.

In human medicine, HPs are characterized by progressive weakness and spasticity of the lower limbs due to distal axonopathy or progressive degeneration of the upper corticospinal motor neurons^5,6^. The onset of HPs can range from early childhood to adulthood, although the specific timing of the onset of the first signs can be difficult to pinpoint, and it is likely that it is often underestimated^7^. The prevalence of HPs has been estimated to be 1.8/100,000^8^. HPs present a wide range of phenotypic and genotypic features and are considered to be one of the most heterogeneous groups of human diseases^9^. The current clinical classification subdivides HPs into “pure” and “complex” forms. “Pure” forms are defined by marked spasticity of the lower limbs, in the absence of other significant findings, except for moderate urinary and distal vibratory sensation impairment^10^. In contrast, “complex” forms are further associated with different other neurological and non-neurological signs, including seizures, cerebellar ataxia, mental retardation, short stature, retinitis pigmentosa, optic atrophy, hearing impairment, and others^6,7^. The HPs clinical classification is considered to be subjective, with the majority of “complex” forms exhibiting phenotypic overlap with other disorders^7^. The advances of molecular diagnosis driven by the development of next generation sequencing facilitated the identification of over 70 HPs associated genes following an autosomal recessive, autosomal dominant or X-linked MOI^1^. The molecular genetic diagnosis has allowed to further classify the HPs based on MOI and affected genes^1,7^. However, even when classified according to genetics, HPs are observed to overlap with other disorders^9^.

Bovine spastic syndrome (SS) is a progressive, adult-onset neuromuscular disorder^11^ that clinically resembles the human HPs “pure” forms. It affects animals of both sexes and is usually diagnosed between 3 and 7 years of age^11–18^. Additionally, SS has been reported in different breeds including Holstein, Jersey, Brown Swiss, Guernsey, Ayrshire, Simmental, Angus, Shorthorn, Hereford, and in crossbreds^13,19^. In the North American Holstein population, SS showed an annual increase of 0.018% between 1994 and 2014^20,21^. Over the 20-year period analyzed, this equates to a total increase of 0.36%, suggesting that the prevalence of SS, which was 0.2% in 1994, appears to have more than doubled over the period studied^20,21^. SS is clinically characterized by intermittent unilateral or bilateral spasm of the skeletal muscles of the pelvic girdle, including the muscles of the rump^11^. The main clinical findings include spasm accompanied by kyphosis with the hindlimbs extended caudally, occasionally accompanied by spastic flexion of the hindlimbs, with tremor^11^. The duration of the spastic episodes varies from a few seconds to several minutes. Following this, the animal re-acquires its normal status^16^. Furthermore, the disorder can manifest in different forms of severity, with the latest affecting not only the hindlimbs and rump but also the neck muscles and forelimbs^17^. Several studies have suggested that SS is inherited as a monogenic recessive trait or is polygenic, but so far, no causative genetic variant has been identified^22,23^.

Therefore, the objective of this study was to describe the phenotype of a series of SS-affected Holstein cattle and to perform a genomic investigation by whole-genome sequencing (WGS).

## Results

### Clinical findings

**Fig. 1:**
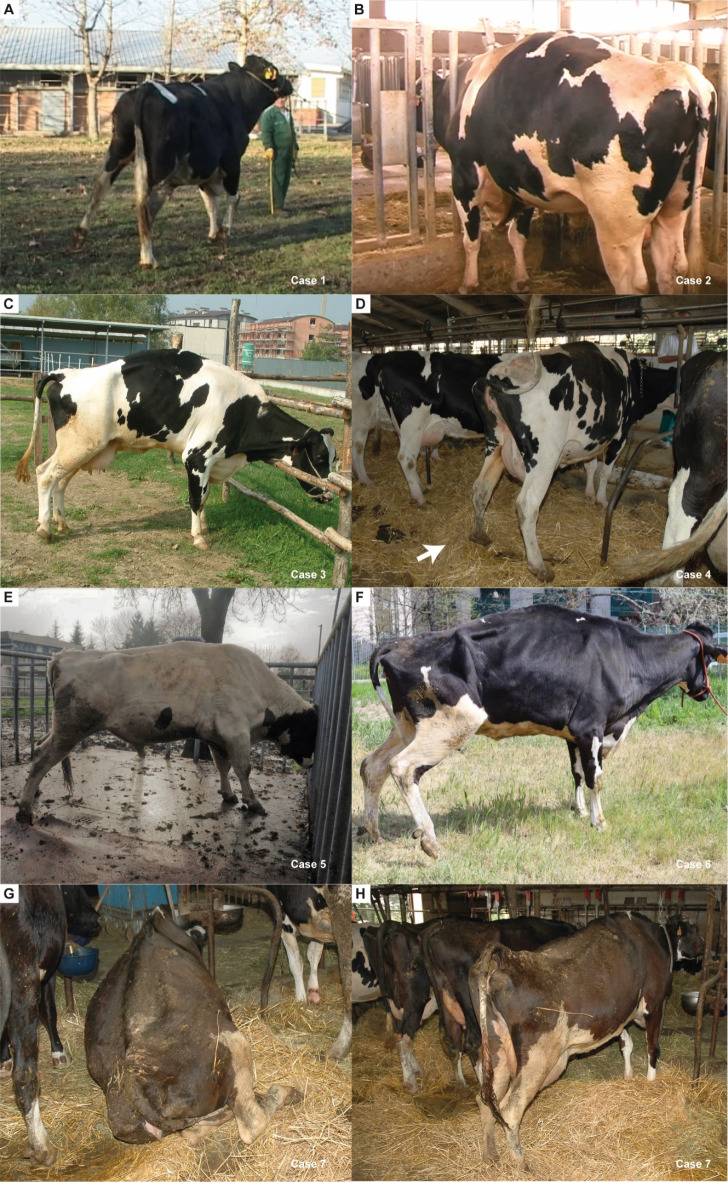
Clinical presentation of spastic syndrome in Holstien cattle. (**A**) Case 1: note the hindlimbs extended in direction latero-caudal bilaterally more pronounced in left side resembling a stretching posture and accompanied by flexion of the hindlimbs during a spastic episode. (**B**) Case 2: note the abnormal posture when standing characterized by kyphosis and contraction of the hindlimbs muscles. (**C**) Case 3: note the abnormal posture characterized by kyphosis and hindlimbs extended in direction latero-caudal bilaterally with slight lift the right hoof during the spastic episode. (**D**) Case 4 (indicated by the arrow): note the abnormal posture characterized by kyphosis and hindlimbs extended in direction latero-caudal bilaterally during the spastic episode. (**E**) Case 5: note the abnormal posture characterized by kyphosis and hindlimbs extended in direction latero-caudal bilaterally resembling a stretching posture. (**F**) Case 6: note the muscle contractions in hindlimbs, rump, trunk, neck and forelimbs accompanied by lifting of the right hindlimb. (**G**) Case 7: Permanent recumbency if not stimulated to stand. (**H**) Case 7: note the abnormal posture when standing characterized by severe kyphosis.

A clinical examination of seven Holstein adult cattle revealed that they were all confirmed to present SS. No anamnestic information was available on possible similar signs or diagnoses in the parents of the SS-affected cattle. In general, all SS cases exhibited spastic muscle contractions accompanied by kyphosis, caudal extension of the hindlimbs, which resembled a stretching posture, spastic episodes accompanied by spastic flexion of the hindlimbs, with tremor and a progressive spastic gate. Both bilateral and unilateral forms were observed (Fig. 1; Supplementary Video 1). A range of additional alterations were identified, but none of these occurred in all cases. The overall clinical findings are presented in Table 1.

**Table 1:** Clinical overview of included bovine spastic syndrome (SS) cases.

Case ID	Sex	Age at recording in years	General clinical examination	Neurologic clinical examination
Case 1	Male	4.5	• Normal skeletal development • Good nutritional status • Normal mental status • Normal major organic functions	• Voluntary standing. • Normal posture when standing (no kyphosis). • Normal muscular tonicity when standing. • Spastic episodes induced by slow walking but not at fast walking. • Spastic episodes characterized by muscle spastic contractions accompanied by kyphosis, hindlimbs extended in direction latero-caudal bilaterally but more pronounced in left side resembling a stretching posture and accompanied by spastic flexion of the hindlimbs, with tremor. • Reduced proprioception of the hindlimbs during the spastic episodes • Spastic episode with a duration of 10–30 s and with a variable interval between episodes. • Progressive spastic gate.
Case 2	Male	4	• Normal skeletal development • Good nutritional status • Normal mental status • Normal major organic functions	• Voluntary standing. • Abnormal posture when standing characterized by kyphosis, wide-stance and hindlimbs extended in direction latero-caudal bilaterally. • Slightly increase muscular tonicity when standing. • Spastic episode induced by standing position, walking and when the animal change from the recumbency to the standing position. • Spastic episodes characterized by muscle spastic contractions accompanied by kyphosis, hindlimbs extended in direction latero-caudal bilaterally resembling a stretching posture and accompanied by spastic flexion of the hindlimbs, with tremor. • Spastic episode with a duration of 10–30 s and with a variable interval between episodes. • Progressive spastic gate.
Case 3	Female	6	• Normal skeletal development • Reduced nutritional status • Normal mental status • Reduced appetite and rumination	• Permanent recumbency if not stimulated to stand. • Abnormal posture when standing characterized by kyphosis, wide-stance and hindlimbs extended in direction latero-caudal bilaterally during the spastic episode. • Spastic episode induced by standing position, walking and when the animal change from the recumbency to the standing position. • Spastic episodes characterized by severe muscle spastic contractions in hindlimbs, rump, and face with tremor. • Spastic episode with a duration of more than 30 s and with and interval of some minutes between episode while standing. • Wide-opened eyes during the spastic episodes. • Progressive spastic gate.
Case 4	Female	4.5	• Normal skeletal development • Good nutritional status • Normal mental status • Normal major organic functions	• Voluntary standing. • Abnormal posture when standing characterized by kyphosis, wide-stance and hindlimbs extended in direction latero-caudal during the spastic episode. • Spastic episode induced when the animal change from the recumbency to the standing position. • Spastic episodes characterized by muscle spastic contractions accompanied by kyphosis, hindlimbs extended in direction latero-caudal bilaterally resembling a stretching posture and accompanied by spastic flexion of the hindlimbs, with tremor. • Spastic episode with a duration of 10–30 s and with a variable interval between episodes. • Progressive spastic gate.
Case 5	Male	4.5	• Normal skeletal development • Good nutritional status • Normal mental status • Normal major organic functions	• Voluntary standing. • Normal posture when standing (no kyphosis). • Spastic episodes induced by walking in hard surfaces and by palpating the quadriceps muscle. • Spastic episodes characterized by muscle spastic contractions accompanied by kyphosis, hindlimbs extended in direction latero-caudal bilaterally resembling a stretching posture and accompanied by spastic flexion of the hindlimbs, with tremor. • Spastic episode with a duration of 10–30 s and with a variable interval between episodes. • Progressive spastic gate.
Case 6	Female	6.5	• Normal skeletal development • Reduced nutritional status • Normal mental status • Normal major organic functions	• Permanent recumbency if not stimulated to stand. • Abnormal posture when standing characterized by kyphosis, wide-stance and hindlimbs extended in direction latero-caudal bilaterally during the spastic episode. • Increase muscular tonicity when standing. • Spastic episode induced by small body movements. • Spastic episodes characterized by severe muscle spastic contractions in hindlimbs, rump, trunk, neck, face and forelimbs accompanied by intermittent lifting of the hindlimbs. • Spastic episode with a duration of more than 30 s and with and interval of some minutes between episode while standing. • Wide-opened eyes during the spastic episodes. • Progressive spastic gate.
Case 7	Female	7	• Normal skeletal development • Reduced nutritional status • Normal mental status • Reduced appetite and rumination	• Permanent recumbency if not stimulated to stand. • Abnormal posture when standing characterized by severe kyphosis, wide-stance and hindlimbs extended in direction latero-caudal bilaterally during the spastic episode. • Increase muscular tonicity when standing. • Spastic episode induced by small body movements, urination, and change from the recumbency to the standing position. • Spastic episodes characterized by severe muscle spastic contractions in hindlimbs, rump, trunk, neck, face and forelimbs accompanied by intermittent lifting of the right hindlimb. • Spastic episode with a duration of more than 30 s and with and interval of some minutes between episode while standing. • Progressive spastic gate.

## Histopathological findings

**Fig. 2:**
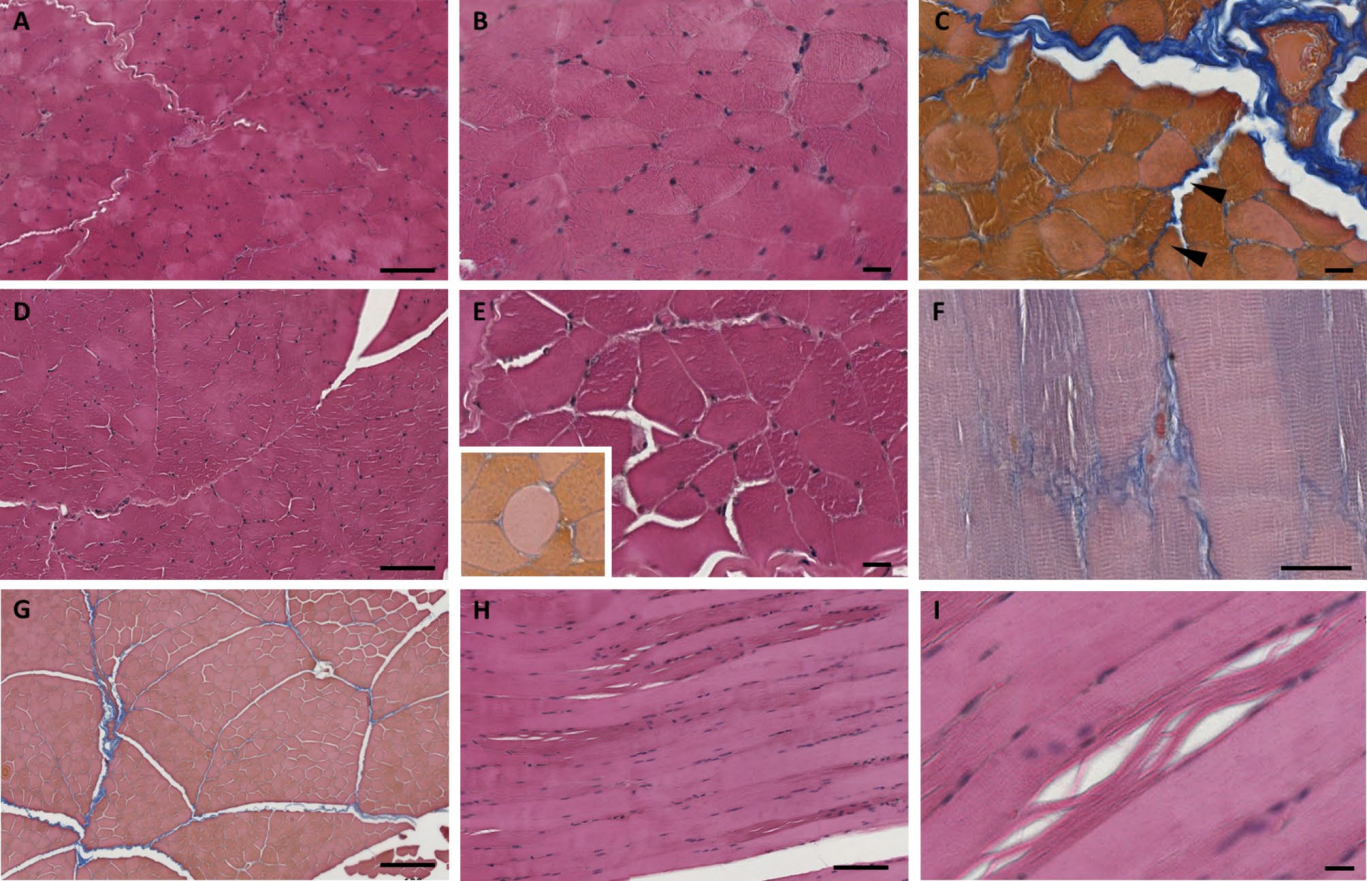
Histological staining on muscle *semimembranosus* (**A-C**), muscle *triceps brachii* (**D-F**) and muscle *quadriceps femoris* (**G-I**) sections from case 6 affected by spastic syndrome. Transversal cross sections from muscle biopsies were stained with H&E (**A**,** B**,** D**,** E**) or with Azan–Mallory method (**C**,** G**), and inset of (**E**) where a round-shape degenerative fiber is shown. Longitudinal sections from muscle biopsies were stained with H&E (**H**,** I**) or with Azan–Mallory methods (**F**). Scale bars 100 μm.

Structural or morphological abnormalities of skeletal muscle fibres were investigated for both in hindlimb and forelimb muscles (Fig. 2). In transversal cross sections, the muscles appeared very similar to healthy muscle. Increased fibre size variability generalized hypotrophy/atrophy characteristics, centrally nucleated myofibers and active skeletal muscle regeneration were not found. Pale round-shaped fibres, presumably close to necrosis (Fig. 2E, inset) were very rare.

Perimysial and endomysial fibrosis, was observed using Azan-Mallory staining. A moderate layer of perimysial fibrosis was found (Fig. 2C), while the generally mild endomysial fibrosis become thick in some areas, as observed in both transverse (Fig. 2C, arrowheads) and longitudinal sections (Fig. 2F). In longitudinal sections, damaged fibres can also be observed showing mall groups of disordered and dark-stained myofibrils, probably due to the severe muscle hyperextension and the increased muscular tonicity when standing.

### Genetic analysis

The seven genomes of the SS-affected cattle were sequenced with an average of 21-fold coverage using short-read WGS technology, and a total of 2.2 Mb in five kb-sized autosomal regions of shared homozygosity were detected (Supplementary file 4). These genomic regions were subjected to a visual using the Integrative Genomics Viewer (IGV), but no obvious structural variants were identified. Moreover, the mean genomic inbreeding coefficient of the seven SS-affected cattle was 0.15 (S.D. ± 0.04) (Supplementary file 5), which is within the normal range for the Holstein breed^24^.

#### A likely pathogenic recessive variant in TOR3A

Assuming a simple recessive MOI, the WGS data were filtered for homozygous coding variants privately present in the seven SS-affected Holstein cattle. However, no single-nucleotide or small indel variants common to all cases were identified. These results suggest that a single shared recessively inherited variant is unlikely to explain the development of SS in Holstein cattle.

Subsequent filtering for homozygous variants only present in the genomes of the individual cases using a global cohort of 5571 bovine control genomes enabled the identification of a private homozygous variant affecting a candidate gene for case 7. The results of the different filtering steps, assuming a recessive inheritance and considering each case individually, are shown in Table 2.

**Table 2:** Results of filtering for possible recessive variants, assuming a homozygous genotype and considering each bovine spastic syndrome (SS) case individually.

Case ID	All variants	Private variants in the SS case using 1031 cattle genome controls	Private protein changing variants in the SS case using 1031 cattle genome controls	Remaining protein-changing private variants using a global control cohort of 4540 cattle genomes and subsequent IGV inspection	NMD candidate gene
Case 1	2,929,327	2,320	29	2	None
Case 2	2,850,131	846	3	0	None
Case 3	2,912,214	1,458	16	0	None
Case 4	2,771,292	945	1	0	None
Case 5	2,126,037	742	9	1	None
Case 6	2,874,923	623	3	0	None
Case 7	2,793,384	1,713	19	1	*TOR3A*

**Fig. 3:**
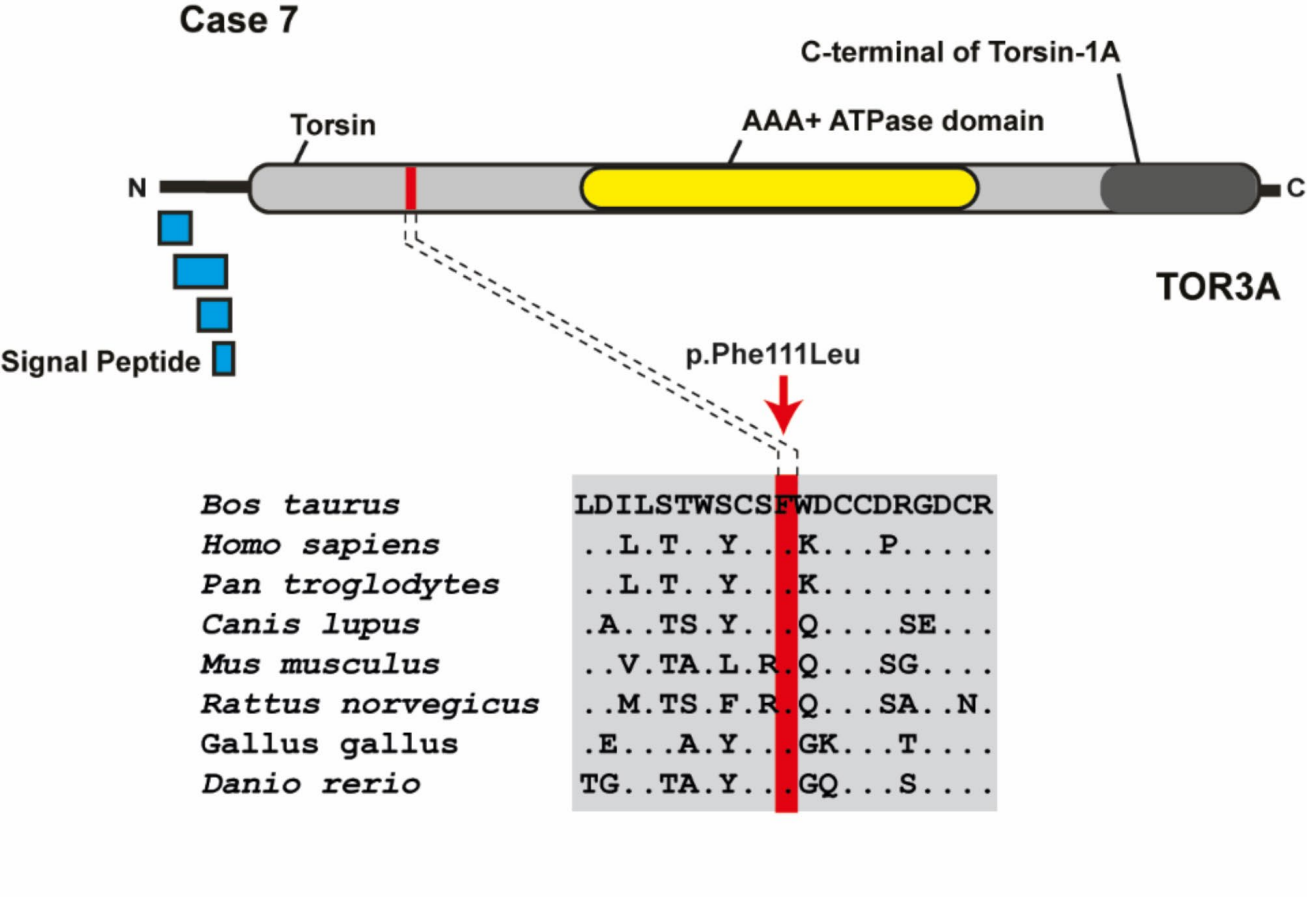
A candidate causal recessive variant in *TOR3A* in Holstein cattle with spastic syndrome. Schematic representation of the bovine TOR3A protein and its functional domains. Multiple sequence alignment of the TOR3A protein encompassing the region of the missense variant found in case 7 demonstrates evolutionary conservation across species.

In case 7, the identified homozygous missense variant in exon 2 of *TOR3A* (Chr16: g.60424685T > C; c.58G > T) was predicted to be deleterious and therefore classified as likely pathogenic. The variant exchanges the encoded amino acid of TOR3A at position 111 (p.Phe111Leu), which is located in the torsin region (Fig. 3). Furthermore, the phenylalanine-to-leucine substitution affects a highly conserved residue.

Subsequently, the *TOR3A* variant was genotyped using the SWISScow array in a population control cohort without any phenotypic records comprising Holstein and Fleckvieh cattle of Switzerland. It was verified that the deleterious allele is present in both populations, with varying low allele frequencies, but not in the homozygous state (Table 3). Furthermore, within the sequenced control genomes cohort, the variant *TOR3A* allele was identified in the heterozygous state in Red Danish, but absent in all other cattle. Considering the global Holstein cohort population^25^, the allelic frequency was estimated to be 1.69% and in the Swiss Holstein population it was 0.61% (Table 3).

**Table 3:** Occurrence of the missense variant in *TOR3A* in different cattle populations.

	Var/Var	Ref/Var	Ref/Ref	Allelic frequency
Global Holstein cattle^a^	1*	40	1209	1.69%
Swiss Holstein^b^	0	71	5785	0.61%
Swiss Fleckvieh^b^	0	6	901	0.33%
Red Danish^a^	0	4	69	2.7%
Sequenced control cattle genomes from other breeds^a^	0	0	4285	0%

*Case 7; ^a^sequenced control genomes cohort form the 1000 Bull Genome Project^25^; ^b^genotyping data from the SWISScow array.

#### Seven candidate causal dominant variants of uncertain significance

**Fig. 4:**
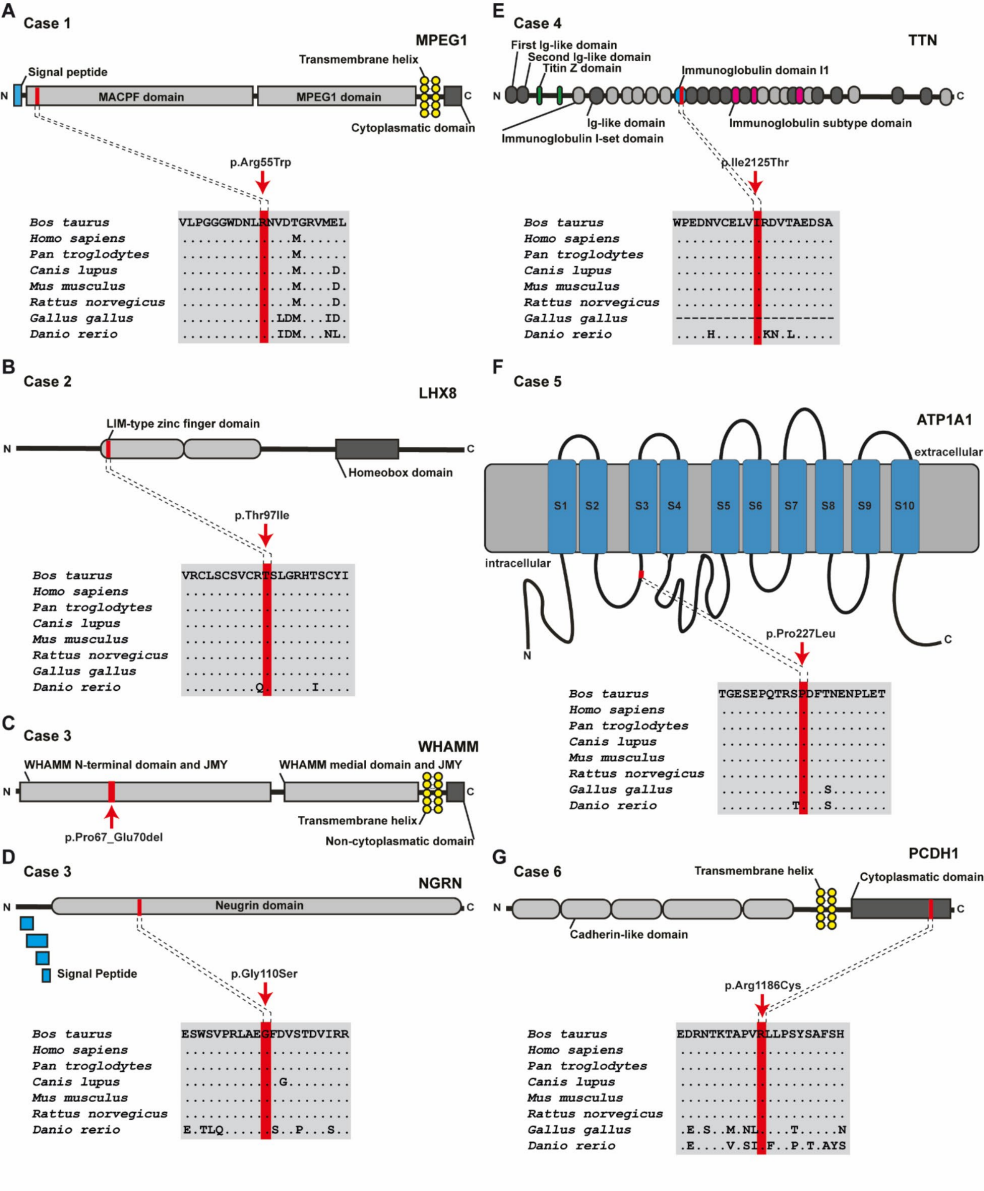
Seven candidate causal dominant variants for spastic syndrome in Holstein cattle. (**A**) Schematic representation of the bovine MPEG1 protein and its functional domains. Multiple sequence alignment of the MPEG1 protein encompassing the region of the missense variant found in case 1 demonstrates complete evolutionary conservation across species. (**B**) Schematic representation of the bovine LHX8 protein and its functional domains. Multiple sequence alignment of LHX8 protein encompassing the region of the missense variant found in case 2 demonstrates complete evolutionary conservation across species. (**C**) Schematic representation of the bovine WHAMM protein and its functional domains. Note that the WHAMM deletion found in case 3 is located in the WHAMM N-terminal domain of the protein (red line and arrow). (**D**) Schematic representation of the bovine NGRN protein and its functional domains. Multiple sequence alignment of the NGRN protein encompassing the region of the missense variant found in case 3 demonstrates complete evolutionary conservation across species. (**E**) Schematic representation of the bovine TTN protein and its functional domains. Multiple sequence alignment of the TTN protein encompassing the region of the missense variant found in case 4 demonstrates complete evolutionary conservation across species. (**F**) Schematic representation of the bovine ATP1A1 protein and its functional domains. Multiple sequence alignment of the ATP1A1 protein encompassing the region of the missense variant found in case 5 demonstrates complete evolutionary conservation across species. (**G**) Schematic representation of the bovine PCDH1 protein and its functional domains. Multiple sequence alignment of the PCDH1 protein encompassing the region of the missense variant found in case 6 demonstrates complete evolutionary conservation across species.

Assuming a dominant MOI, WGS data were filtered for heterozygous private variants that were present individually in each case and absent in all controls. This approach revealed variants in different NMD candidate genes for six SS-affected cattle (cases 1–6). The results of the different filtering steps searching for dominant variants as cause of bovine SS are shown in Table 4. These nine heterozygous variants were further analysed for their predicted effect on the encoded protein. From these, seven variants affecting seven different genes were exclusively present in the genome of each case and predicted to be deleterious. Six of the variants were missense variants affecting highly conserved residues, while one was a small deletion. The identified variants are presented in Table 5; Fig. 4.

**Table 4:** Results of filtering for dominant variants, assuming a heterozygous genotype and considering each bovine spastic syndrome (SS) case individually.

Case ID	All variants	Private variants in the SS case using 1031 cattle genome controls	Private protein changing variants in the SS case using 1031 cattle genome controls	Remaining protein-changing private variants using a global control cohort of 4540 cattle genomes and subsequent IGV inspection	NMD candidate gene(s)
Case 1	4,665,254	468	2	1	*MPEG1*
Case 2	4,794,525	1,107	10	3	*LHX8*
Case 3	4,664,808	4,007	13	5	*WHAMM*,* NGRN*
Case 4	4,868,152	3,476	29	7	*TTN*
Case 5	6,278,631	1,087	10	2	*ATP1A1*,* RNASE11*
Case 6	4,620,348	937	3	3	*PCDH1*,* SERTAD2*
Case 7	4,458,121	2,708	18	2	None

**Table 5:** List of detected candidate variants in seven bovine spastic syndrome (SS) cases.

Case ID	Gene	Uniprot ID/OMIM ID	Gene function/ Associated disorder ^a^/ expression in humans	Variant type	Variant features (genomic, cDNA, protein)
Case 1	*MPEG1*	Q2M385/OMIM619223	Central role in antigen cross-presentation in dendritic cells by forming a pore in antigen-containing compartments. Immunodeficiency 77. Highly expressed in nervous system.^b^	Heterozygous missense	Chr15:g.82344225G > A c.163 C > T p.Arg55Trp
Case 2	*LHX8*	Q68G74/OMIM604425	Transcription factor involved in differentiation of certain neurons and mesenchymal cells. Highly expressed in brain.^c^	Heterozygous missense	Chr3.g.69655510G > A c.290 C > T p.Thr97Ile
Case 3	*WHAMM*	Q8TF30/OMIM612393	Acts as a nucleation-promoting factor that stimulates Arp2/3-mediated actin polymerization both at the Golgi apparatus and along tubular membranes. Highly expressed in nervous system.^b^	Heterozygous small deletion	Chr21:g.23082172CGCCCGAGCCCGA > C c.199_210delCCCGAGCCCGAG p.Pro67Glu70del
	*NGRN*	Q9NPE2/OMIM616718	Plays an essential role in mitochondrial ribosome biogenesis. Highly expressed in neuromuscular system. ^b^	Heterozygous missense	Chr21: g.21640669G > A c.328G > A p.Gly110Ser
Case 4	*TTN*	Q8WZ42/OMIM188840	Dilated cardiomyopathy; congenital myopathy with cardiomyopathy; muscular dystrophy limb-girdle; myopathy myofibrillar with early respiratory failure; tardive tibial muscular dystrophy. Highly expressed in neuromuscular system. ^b^	Heterozygous missense	Chr2.g.18094040T > C c.6374T > C p.Ile2125Thr
Case 5	*ATP1A1*	P05023/OMIM182310	Hereditary spastic paraplegia, Charcot-Marie-Tooth disease; hypomagnesemia, seizures, and impaired intellectual development. Highly expressed in neuromuscular system. ^b^	Heterozygous missense	Chr3:g.26902947G > A c.680 C > T p.Pro227Leu
Case 6	*PCDH1*	Q08174/OMIM603626	Mediate calcium-dependent cell-cell adhesion. Highly expressed in nervous system.^b^	Heterozygous missense	Chr7:g.52935731G > A c.3556 C > T p.Arg1186Cys
Case 7	*TOR3A*	Q9H497/OMIM607555	Dystonia.^d^ Highly expressed in neuromuscular system.^b^	Homozygous missense	Chr16:g.60424685T > C c.58G > T p.Phe111Leu

ID, identification; OMIM, Online Catalog of Human Genes and Genetic Disorders; ^a^, if available; ^b^, Kelleher et al.^30^; ^c^, Uhlén et al.^28^; ^d^, Naismith et al.^29^.

In case 1, the identified private heterozygous variant was a missense variant in exon 1 of *MPEG1* that exchanges the encoded amino acid of MPEG1 at position 55, located at the membrane attack complex component/perforin (MACPF) domain (Fig. 4A). In case 2, the identified variant was a missense variant in exon 4 of *LHX8* and exchanges the encoded amino acid of LHX8 at position 97, located at the LIM-type zinc finger domain (Fig. 4B). In case 3, two candidate variants were identified. One was a 12 bp deletion in exon 1 of *WHAMM* (Fig. 4C). The second variant was a missense variant in exon 3 of *NGRN* that exchanges the encoded amino acid of NGRN at position 110, located at the neugrin domain (Fig. 4D). In case 4, the identified variant was a missense variant in exon 28 of the *TTN* that exchanges the encoded amino acid of TTN at position 2125, located at the immunoglobulin subtype domain (Fig. 4E). In case 5, the identified variant was a missense in exon 7 of *ATP1A1* that exchanges the encoded amino acid of ATP1A1 at position 227, located at the alpha subunit of Na(+)/K(+)-ATPase domain (Fig. 4F). Finally, in case 6, the identified variant was a missense variant in exon 5 of *PCDH1* that exchanges the encoded amino acid of PCDH1 at position 1186 located at the cytoplasmatic domain (Fig. 4G). Although all these private coding variants were predicted to be deleterious (Supplementary file 6), they were classified as variants of uncertain significance due to their heterozygous state.

## Discussion

This study was performed on seven Holstein cattle exhibiting a common clinical presentation of spastic syndrome. This presentation was characterized by muscle contractions with kyphosis, caudal extension of the hindlimbs resembling a stretching posture, spastic episodes with spastic flexion of the hind limbs, tremor, and a progressive spastic gate. The heterogeneous group of human NMDs, in particular the HPs, share some similarities with the clinical presentation of bovine SS, including progressive weakness and spasticity^7,26^. Using a comparative approach and the hypothesis of Mendelian causes, similar to numerous inherited NMD forms in humans, we have for the first time performed a whole genome-wide search for possible causal variants in cattle with SS. Thereby, we prioritised protein-changing variants in bovine orthologs of known human NMD-related genes when analysing the genome of SS-affected cattle.

In bovine medicine, SS is frequently misdiagnosed as spastic paresis, another NMD known in cattle, or vice versa. This is due to the fact that both conditions have a similar terminology, which can lead to misdiagnosis. This may also explain why the genetic cause of the diseases, if inherited, is not clear. However, the two bovine forms of NMDs exhibit strikingly different clinical manifestations. As demonstrated in our study, SS is a late-onset progressive NMD, characterized by spastic episodes. In contrast, spastic paresis is typically an earlier-onset NMD, characterized by hyperextension of the hindlimb due to permanent spasm, primarily affecting the gastrocnemius and/or quadriceps muscles^27^.

A simple recessive Mendelian inheritance has previously been hypothesised for SS in cattle as well as a more complex polygenic inheritance^15,16,23^. Alternatively, it has been speculated that SS may represent a group of diseases with different causes but a similar clinical presentation^17^. Here, we report eight protein-changing variants affecting eight different genes in seven SS-affected cattle that are associated with NMDs in humans and/or with high expression in neuromuscular tissue (Table 5)^28–30^. For the first time, WGS genomic analysis of SS-affected cattle showed that this neuromuscular syndrome in cattle can possibly be explained by a broad genetic heterogeneity, including both recessive and dominant variants. Furthermore, the possibility of both monogenic and potential digenic inheritance has also been presented. The types of variants that may be in SS were found to range from SNVs to small indels. The discovery that SS in cattle is likely to be caused either by deleterious recessive alleles or by different dominant variants offers new insights into the understanding of SS and the function of the genes involved. Nevertheless, a more comprehensive clinical and histological investigation of bovine SS cases, in conjunction with further genotyping of candidate causal variants and functional experiments to evaluate the variants of uncertain significance, is necessary to elucidate the association between the identified genetic variants and this neuromuscular syndrome.

A recent study has suggested that SS in Holstein cattle may be associated with genomic regions of interest on chromosomes 7 and 9^23^. In addition, the same study has proposed that *Fig. 4*,* FYN* and *CACNA1A* are candidate genes for bovine SS^21^. However, neither shared regions of homozygosity nor candidate variants in these genes were observed in the SS cases presented in our study. Consequently, it appears less probable that variants in the aforementioned genes can account for all cases of SS in Holstein cattle.

In our study, assuming a recessive MOI we identified a homozygous missense variant in *TOR3A* in one case. The *TOR3A* gene encodes the torsin-3 A protein that is predicted to enable ATP binding activity and is highly expressed in the neuromuscular system in adults^31,32^. The main domain of this protein is the torsin-A that represents an essential AAA + ATPase (ATPases associated with a variety of cellular activities) found in the endoplasmic reticulum and nuclear envelope of higher eukaryotes^31,33^. A loss of function of torsin-A can result in the development of the torsion dystonia (OMIM128100) that has been reported to be dominantly inherited. Pathogenic variants in *TOR1A* have been identified and associated with this type of dystonia that is further characterized by tremor, hypotonia and Writter’s cramp^29,34,35^. Additionally, isolated focal dystonia may occur^34^. Some clinical features shown in human torsion dystonia could be identified also in case 7. Given the similarity between the two proteins and the evidence that *TOR1A* and *TOR3A* interact^29^, it is reasonable to suggest that the identified recessive variant in bovine *TOR3A* may be causative for SS in cattle.

It is proposed that five heterozygous protein-changing variants located in seven different genes (*MPEG1*, *LHX8*, *TTN*, *ATP1A1*, *PCDH1*) involved in neuromuscular function may be responsible for SS in six Holstein cattle, assuming dominant MOI. We postulated that *MPEG1* might be a potential candidate gene, hypothesizing that the identified missense variant could result in an immunologic form of NMD as *MPEG1* plays a central role in antigen cross-presentation in dendritic cells^36^. Immune-mediated NMDs have been described in humans^37,38^ and represent a broad spectrum of disorders affecting the nervous system, including multiple sclerosis and chronic inflammatory demyelinating polyneuropathy^39–41^. To confirm the causality of the identified variant in *MPEG1* and its association with the observed phenotype, a more detailed study of the case, including immunological and functional experiments, would have been required. *LHX8*^32,42^ mutant mice exhibit several abnormalities in the nervous system including abnormal striatum morphology and abnormal neuron morphology including alterations in neuron differentiation, brain interneuron and GABAergic neuron morphology as well as in cholinergic neuron morphology^43,44^. In mammals, such abnormalities have been associated with several forms of NMD, neurodevelopmental and/or neurodegenerative disorders^44–47^. Given the clinical signs observed in the SS-affected cattle and the function of *LHX8* and the associated disorders in other mammal species, it is plausible that the identified variant could be causal and therefore explain the observed phenotype. In humans, heterozygous variants in *TTN* are associated, within others, with tardive tibial muscular dystrophy (OMIM600334), a late-onset disease characterized by weakness and atrophy of the anterior compartment of the lower leg, specifically the tibialis anterior muscle^48,49^. Although human tardive tibial muscular dystrophy does not completely overlap with the clinical presentation of bovine SS, some similarities can be identified, such as the onset, muscle weakness and atrophy, and the fact that it mostly affects the lower limbs, which correspond to the hind limbs in cattle. Therefore, it is reasonable that the identified variant in *TTN* could explain SS in case 4^50–52^. In human medicine, heterozygous variants affecting *ATP1A1* have been associated with different disorders including an early-onset form of HP characterized mainly by spastic gait with pyramidal signs at the lower limbs^53^. Considering that bovine SS is clinically similar to HP, the identification of the variant in a gene known to be a candidate gene for HP strengthens the similarities between these two diseases not only at the phenotypic but also at the molecular level. There is limited knowledge on the role of *PCDH1* in neuromuscular disorders, but it has been shown that in mammals, clustered protocadherin genes are molecular “identifiers” fundamental for encoding the cell surface that allows neural “self/non-self” discrimination^54^. Moreover, studies in humans demonstrate that variants in the clustered protocadherin genes are associated with several neurodevelopmental disorders^55^. In light of the expression and function of *PCDH1* as well as its association with nervous system disorders in humans, the variant in bovine *PCDH1* may be the cause of SS in one case.

Furthermore, in one case, two potential heterozygous protein-changing candidate variants affecting *WHAMM* and *NGRN* were identified. *WHAMM*^56,57^ recruits and activates the Arp2/3 complex for actin assembly at sites of autophagosome formation on the endoplasmic reticulum^58^. A novel group of inherited congenital neurodevelopmental and adult-onset neurodegenerative disorders due to defects of autophagy machinery or closely related proteins has recently been described^59^. To date, *WHAMM* has not been associated with this group of disorders, but some genes (e.g., *LAMTOR2*, *LRRK2*, *MTM1*, *TBCK*) with a function in regulating autophagy induction, similar to *WHAMM*, have been associated with congenital neurodevelopmental and adult-onset neurodegenerative disorders^59^. The identification of a protein-changing variant in *WHAMM* with a possible association to SS provides new insights of the gene function and add it to the list of candidate genes due to defects of autophagy. The second heterozygous variant identified in a single case affected the *NGRN* that plays an essential role in mitochondrial ribosome biogenesis and neuronal regeneration and is highly expressed in the neuromuscular system in adults^32,60,61^. However, no phenotype-related data are available. Only case 4 was heterozygous for both the *WHAMM* and *NGRN* variants with the absence of further variant carriers within the control cohort. We therefore hypothesized that possibly heterozygosity for both variants in *WHAMM* and *NGRN* would be required and consequently, a combined or additive effect of both variants may be a plausible explanation for the SS phenotype. It is noteworthy that digenic forms of inheritance involving *WHAMM* and *NGRN* have never been reported. However, with the evidence presented in the current study, it cannot be excluded that the presence of the heterozygous variant in *WHAMM* or alternatively in *NGRN* itself might be causative. Future research will be needed to provide valuable additional insights into the biological effects of the identified *WHAMM* and *NGRN* variants.

Considering the known function of *TOR3A*, *MPEG1*, *LHX8*, *TTN*, *ATP1A1*, *PCDH1*, *WHAMM* and *NGRN* genes, the rarity of the associated candidate variants, and the outcome of the in silico effect prediction, the identified candidate variants in these genes were assumed to represent plausible candidate causal variants for bovine SS. Therefore, our WGS approach was able to provide a potential molecular genetic diagnosis in all SS cases studied. The efficiency of WGS for genetic diagnosis in cattle has recently been investigated for a fatal congenital syndrome in cattle with a diagnostic rate of approximately 50%^62^. Although the results obtained in the current study are based on a smaller number of affected animals, it is possible that the diagnostic rate for bovine SS may be higher.

A limitation of our study was the absence of information on the clinical history of the parents as well as their biological material. These would have been necessary to provide stronger evidence for the causality of the identified variants with an expected dominant MOI, as it would have been possible to investigate the origin of the variants precisely: either the variants arose de novo in early embryonic development, were inherited from mosaic germline of one of the parents or were inherited from an SS-affected parent. For example, it would have been possible to exclude that the identified deleterious alleles did not segregate in healthy parents, or that they did segregate, but in that case the parent was also affected by SS. In addition, genotyping of future SS cases for the recessive *TOR3A* variant as well as of the heterozygous variants in *WHAMM* and *NGRN* will be important to evaluate possible causality of these variant alleles. It should also be noted that the genome of case 7 had lower coverage compared to the other cases and therefore heterozygous variants in the genome may have been missed. Finally, further experimental studies are needed to functionally validate the postulated causality of the herein identified candidate variants for bovine SS.

Here, we propose eight protein-changing variants as possible explanations for SS in cattle. We also report for the first-time phenotype-gene associations for *TOR3A1*, *WHAMM*, and *NGRN*, providing new candidate genes for NMDs and neurodegenerative disorders in mammalian species. Finally, our study highlights that the molecular genetics of inherited disorders in well-phenotyped large animals, such as cattle, is a valuable model system for studying fundamental aspects of gene function.

## Methods

### Ethics statement

All examinations were carried out after obtaining written informed owner’s consent and in accordance with local laws, regulations, and ethical guidelines. The Animal Welfare Committee (CoBA) of the University of Bologna was consulted and it was considered that ethical approval was not required as the study was not experimental but part of clinical and pathological veterinary diagnostics. The animals were handled in accordance with good veterinary practice. The animals were handled in accordance with Council Directive 98/58/EC and the Italian National Animal Welfare Plan (Piano Nazionale Benessere Animale 2024 - PNBA). All animals were referred to the Ruminant Clinic of the University of Bologna for veterinary diagnostic purposes and not for research. The cases were used retrospectively for the preparation of this manuscript.

### Animals and phenotypical investigation

This study included seven Holstein adult cattle suspected to be affected by SS that were submitted to the Department of Veterinary Medical Sciences, University of Bologna, Italy. Three animals were sires and four were cows. The mean age of the cases at referral was of 5.3 years (S.D.±1.1). The seven animals underwent a complete clinical examination. In addition, case 6 showed worsening of the general condition related to the neuromuscular disorder and was euthanized because of welfare reasons. For the euthanasia, the animal was first sedated with 1.5 mg/kg of xylazine intravenous (IV) and then euthanized with 200 mg/kg of sodium thiopental IV. The animal was subsequently submitted for necropsy and histologic examination. Muscle *semimembranosus*, muscle *triceps brachii* and muscle *quadriceps femoris* were fixed in buffered neutral paraformaldehyde at 4 °C, washed in phosphate-buffered saline and dehydrated through a graded series of ethanol. Samples embedded in paraffin were cut at 5 μm and stained with Hematoxylin and Eosin (H&E), or Azan–Mallory method, specific for detection of collagen fibers. Muscle sections were scanned with a semiautomatic microscope equipped (D-Sight v2, Menarini Diagnostics, Florence, Italy) with a computer.

### DNA extractions

Genomic DNA was obtained from the affected animals (EDTA blood samples) using Promega Maxwell RSC DNA system (Promega, Dübendorf, Switzerland).

### Whole-genome sequencing and variant calling

A whole-genome sequencing data was generated using the Illumina NovaSeq6000 (Illumina Inc., San Diego, CA, USA) on the genomic DNA extracted from the seven SS cases. The sequenced reads were mapped to the ARS-UCD1.2 reference genome, resulting in an average read depth of approximately 24.7× in case 1, 27× in case 2, 23.8× in case 3, 18.8× in case 4, 22.1× in case 5, 18× in case 6, and 13.7× in case 7 and single-nucleotide variants and small indel variants were called^63^. The applied software and steps to process fastq files into binary alignment map (BAM) and genomic variant call format (GVCF) files were in accordance with the 1000 Bull Genomes Project processing guidelines of run 7^25^, except for the trimming, which was performed using fastp^64^. The resulting individual GVCF files were merged into one large variant call format (VCF) file using CombineGVCFs and CatVariants of gatk v3.8^65^. SNVs and insertions and deletions were called using GenotypeGVCF of gatk version 3.8 and were given a quality label based on the best practice recommendations in GATK using the VariantFiltration of gatk v3.8^65^. The effects of the above variants were functionally evaluated with snpeff v4.3^66^, using the NCBI Annotation Release 106 (https://www.ncbi.nlm.nih.gov/genome/annotation_euk/Bos_taurus/106/; acceded on 20 September 2022). This resulted in the final VCF file, comprising individual variants and their functional annotations as described before^67^. To identify private variants, we compared the genotypes of the cases with 1031 cattle genomes of different breeds sequenced as part of the ongoing Swiss Comparative Bovine Resequencing project. All its data are available in the European Nucleotide Archive (project accession number PRJEB18113; Supplementary file 1). Regarding the MOI three different scenarios were hypothesized: (i) autosomal recessive mode of inheritance common to all SS cases, (ii) autosomal recessive mode of inheritance considering each SS case individually, or (iii) dominant mode of inheritance considering each SS case as an isolated event. IGV^68^ software version 2.0 was used for visual evaluation of genome regions containing potential candidate genes.

### Runs of homozygosity

A genome-wide search for homozygous regions shared by the Holstein cases was performed using the R package detectRUNS v.0.9.6^69^. In addition, the genomic inbreeding coefficient was calculated and compared with the mean genomic inbreeding coefficient obtained from the analysis of 360 control Holstein genomes from the Swiss Comparative Bovine Resequencing project using using the R package detectRUNS v.0.9.6^69^.

### Occurrence of variants in a global control cohort

The comprehensive variant catalogue from run 9 of the 1000 Bull Genomes Project was available to investigate the allelic distribution of variants within a global control cohort^25^. The full dataset includes 5116 bovine genomes, including 576 from the Swiss Comparative Bovine Resequencing Project, from a wide variety of more than 130 breeds. Within the dataset, there were 1209 purebred Holstein cattle allowing for the exclusion of variants common to these breeds.

Moreover, the p.Phe111Leu missense variant in *TOR2A* was genotyped using the high density Axiom Microarray Genotyping Technology (SWISScow). The array has been developed under the umbrella of the Swiss routine genomic system, which has genotyped several thousand animals from current Swiss dairy population since 2020 and contains 308,512 variants, including common “routine markers” considered for genomic selection, as well as more than 50 variants that cause known bovine Mendelian disorders. Evaluation of the prevalence of this deleterious allele in Swiss Fleckvieh, and Swiss Holstein was performed using a total of 6763 cattle.

### In silico assessment of the molecular consequences

PredictSNP1^70^ was used to predict the biological consequences of the candidate SNVs and Provean^71^ was used to predict the biological consequences of the candidate SNVs and small indels. GnomAD was used to predict the probability of a gene being intolerant (pLI)^72^.

### Candidate gene and candidate variant classification

The term “candidate gene” was used to describe genes based on function and/or associated NMD-related phenotypes in mammalian species. The term candidate variant was used to describe variants that took into account the affected gene function and/or associated phenotype in mammalian species, rarity, and the predicted effect of the variant on the encoded protein, with at least one tool predicting it to be deleterious. The variants were further classified according to the Standards and Guidelines for the Interpretation of Sequence Variants^73^. All sequence accessions used for the candidate variants are listed in Supplementary files 2 and 3.

## Electronic supplementary material

Below is the link to the electronic supplementary material.

Supplementary Material 1

Supplementary Material 2

Supplementary Material 3

Supplementary Material 4

Supplementary Material 5

Supplementary Material 6

Supplementary Video 1

## Data Availability

Sequence data that support the findings of this study have been deposited in the European Nucleotide Archive within the project PRJEB18113 and the following sample accessions: SAMEA6528905, SAMEA6528906, SAMEA6528907, SAMEA6528908, SAMEA6528909, SAMEA111531539, and SAMEA19876918.
