# Supplementary material for: Whole genome sequencing reveals candidate causal genetic variants for spastic syndrome in Holstein cattle
Source: Sci Rep. 2024 Dec 28;14:31188. doi: 10.1038/s41598-024-82446-z (PMC11682090; doi:10.1038/s41598-024-82446-z)
Supplement: Supplementary file 1 — Supplementary Material 1 [file 41598_2024_82446_MOESM1_ESM.docx]

**Additional file 1:** Sample IDs from whole-genome sequencing samples stored in the European Nucleotide Archive.

| **ID** | **BAM ID** | **Breed** | **EBI Project ID** | **EBI Sample ID** | **Average Coverage** |
| --- | --- | --- | --- | --- | --- |
| Case 1 | RM2815 | Holstein | PRJEB18113 | SAMEA6528905 | 24.7 |
| Case 2 | RM2817 | Holstein | PRJEB18113 | SAMEA6528906 | 27.0 |
| Case 3 | RM2821 | Holstein | PRJEB18113 | SAMEA6528907 | 23.8 |
| Case 4 | RM2824 | Holstein | PRJEB18113 | SAMEA6528908 | 18.8 |
| Case 5 | RM2833 | Holstein | PRJEB18113 | SAMEA6528909 | 22.1 |
| Case 6 | RM4725 | Holstein | PRJEB18113 | SAMEA111531539 | 18.0 |
| Case 7 | SS013 | Holstein | PRJEB18113 | SAMEA19876918 | 13.7 |
