# Supplementary material for: Whole genome sequencing reveals candidate causal genetic variants for spastic syndrome in Holstein cattle
Source: Sci Rep. 2024 Dec 28;14:31188. doi: 10.1038/s41598-024-82446-z (PMC11682090; doi:10.1038/s41598-024-82446-z)
Supplement: Supplementary file 2 — Supplementary Material 2 [file 41598_2024_82446_MOESM2_ESM.docx]

**Supplementary file 2:** Sequence accessions numbers of candidate variants. Chromosome, mRNA and Protein references correspond to the NCBI accessions using ARS-UCD1.2 reference genome and Protein structure references correspond to the UniProt accession of the *Bos taurus* protein.

| **Gene** | **Chromosome** | **mRNA** | **Protein** | **Protein structure** |
| --- | --- | --- | --- | --- |
| *MPEG1* | Chr15: NC 037342.1 | NM_001046464.1 | NP_001039929.1 | Q2KJC3 |
| *LHX8* | Chr3: NC_037330.1 | XM_002686301.5 | XP_002686347.1 | E1BBB7 |
| *TTN* | Chr2: NC_037329.1 | XM_024979882.1 | XP_024835650.1 | A0A4W2F6Z2 |
| *ATP1A1* | Chr3: NC_037330.1 | NM_001076798.1 | NP_001070266.1 | Q08DA1 |
| *PCDH1* | Chr7: NC_037334.1 | XM_024994061.1 | XP_024849829.1 | A0A3Q1M024 |
| *WHAMM* | Chr21: NC_037348.1 | NM_001191456.1 | NP_001178385.1 | E1BD74 |
| *NGRN* | Chr21: NC_037348.1 | NM_001045992.2 | NP_001039457.2 | Q2HJC0 |
| *TOR3A* | Chr16: NC_037343.1 | NM_001080376.1 | NP_001073845.1 | A1A4M8 |
