# Supplementary material for: Whole genome sequencing reveals candidate causal genetic variants for spastic syndrome in Holstein cattle
Source: Sci Rep. 2024 Dec 28;14:31188. doi: 10.1038/s41598-024-82446-z (PMC11682090; doi:10.1038/s41598-024-82446-z)
Supplement: Supplementary file 3 — Supplementary Material 3 [file 41598_2024_82446_MOESM3_ESM.docx]

**Supplementary file 3:** Sequence accession numbers the proteins used to perform the multiple alignment for of the candidate variants.

| **Protein** | **Protein accessions** |
| --- | --- |
| MPEG1 | *Homo sapiens*: NP_001034485.1  *Pan troglodytes*: XP_508450.1  *Canis lupus*: XP_533169.2  *Rattus norvegicus*: NP_001292389.1 |
|  | *Mus musculus*: NP_001361597.1  *Gallus gallus*: XP_003641422.2  *Danio* *rerio*: NP_997902.1 |
| LHX8 | *Homo sapiens*: NP_001243043.1  *Pan troglodytes*: XP_016776373.1  *Canis lupus*: XP_038390822.1  *Rattus norvegicus*: NP_001402020.1  *Mus musculus*: NP_034843.2  *Gallus gallus*: NP_001035556.2  *Danio* *rerio*: NP_001003980.1 |
| TTN | *Homo sapiens*: NP_001254479.2  *Pan troglodytes*: XP 024210409.2  *Canis lupus*: XP_038319332.1  *Rattus norvegicus*: XP_063140776.1  *Mus musculus*: NP_001372637.1  *Danio rerio*: XP_021334746.1 |
| ATP1A1 | *Homo sapiens*: NP_000692.2  *Pan troglodytes*: XP_513679.3  *Canis lupus*: XP_038546393.1  *Rattus norvegicus*: NP_036636.1  *Mus musculus*: NP_001390724.1  *Gallus gallus:* NP_001039292.2  *Danio rerio*: XP_021334746.1 |
| PCDH1 | *Homo sapiens*: NP_115796.2  *Pan troglodytes*: XP_003310925.1  *Canis lupus*: XP_003638961.1  *Rattus norvegicus*: XP_038953281.1  *Mus musculus*: NP_659149.1  *Gallus gallus:* NP_990852.2  *Danio rerio*: XP_009289494.1 |
| NGRN | *Homo sapiens*: NP_001028260.2  *Pan troglodytes*: XP_009428102.2  *Canis lupus*: XP_003434423.1  *Rattus norvegicus*: NP_001029072.2  *Mus musculus*: NP_113552.3  *Danio rerio*: NP_001038718.1 |
| TOR3A | *Homo sapiens*: NP_071766.2  *Pan troglodytes*: XP_514028.5  *Canis lupus*: XP_038526558.1  *Rattus norvegicus*: NP_001009683.1  *Mus musculus*: NP_075630.2  *Gallus gallus:* XP_422270.2  *Danio rerio*: NP_001121738.1 |
