# Supplementary material for: Whole genome sequencing reveals candidate causal genetic variants for spastic syndrome in Holstein cattle
Source: Sci Rep. 2024 Dec 28;14:31188. doi: 10.1038/s41598-024-82446-z (PMC11682090; doi:10.1038/s41598-024-82446-z)
Supplement: Supplementary file 6 — Supplementary Material 6 [file 41598_2024_82446_MOESM6_ESM.docx]

**Supplementary file 6:** Protein prediction of the candidate variants according to PredictSNP and Provean.

| **Gene** | **Variant** | **PredictSNP** | **Provean** |
| --- | --- | --- | --- |
| *MPEG1* | p.Arg55Trp | Deleterious (score: 87%) | Deleterious (score: -6.580) |
| *LHX8* | p.Thr97Ile | Neutral (score: 80%) | Deleterious (score: -3.120) |
| *TTN* | p.Ile2125Thr | Deleterious (score: 87%) | Deleterious (score: -3.461) |
| *ATP1A1* | p.Pro227Leu | Deleterious (score: 50%) | Deleterious (score: -4.822) |
| *PCDH1* | p.Arg1186Cys | Deleterious (score: 61%) | Neutral (score: -1.869) |
| *WHAMM* | p.Pro67_Glu70del | NA | Deleterious (score: -3.293) |
| *NGRN* | p.Gly110Ser | Neutral (score: 63%) | Deleterious (score: -2.719) |
| *TOR3A* | p.Phe111Leu | Deleterious (score: 63%) | Deleterious (score: -3.205) |

NA, not available
